# The Mechanism of Relieving Diarrheal Irritable Bowel Syndrome Using Polyphenols from *Ribes nigrum* L. Based on a Network Pharmacology Analysis and 16S rRNA Sequencing

**DOI:** 10.3390/foods13233868

**Published:** 2024-11-29

**Authors:** Xi Yu, Xiaotian Wang, Xintong Liu, Fangfei Li, Yihong Bao, Yangyang Chai

**Affiliations:** 1 School of Life Sciences, Northeast Forestry University, Harbin 150040, China; 13153579925@163.com (X.Y.); tyanyuuwang@163.com (X.W.); lxt2251115@163.com (X.L.); lifangfei33@163.com (F.L.); baoyihong@163.com (Y.B.); 2 Key Laboratory of Forest Food Resources Utilization of Heilongjiang Province, Harbin 150040, China

**Keywords:** *Ribes nigrum* L. polyphenol, diarrheal irritable bowel syndrome, FoxO signal pathway, 16S rRNA sequencing

## Abstract

Diarrheal irritable bowel syndrome (IBS-D) is a chronic bowel condition that leads to intestinal dysfunction and is typically accompanied by diarrhea, stomach pain, and abdominal distension. *Ribes nigrum* L. polyphenols (RNPs), which are natural plant polyphenols, are the subject of this study, which aims to assess their potential in improving IBS-D and to explore the underlying mechanisms through a network pharmacology analysis and 16S rRNA sequencing. Next, mice models of diarrhea-predominant irritable bowel were established, and the mice with IBS-D were treated with RNPs. The effect of RNPs was then evaluated in terms of body weight, abdominal withdrawal reflex (AWR), Bristol score, fecal water percentage, diluted fecal volume, total intestinal transit time, immune index, histopathological observation, and changes in inflammatory factors. Finally, 16S rRNA sequencing and reverse q-RTPCR were utilized to evaluate the components that mediate the impact of RNPs on IBS-D. It was found that when RNP treatment was administered to mice with IBS-D, they decreased the water content in their stools, raised their immunological scores, and decreased the amount of inflammatory substances in their bodies. Moreover, through 16S rRNA sequencing, it was shown that the RNP treatment increased the relative abundances of Bacteroides, Alloprevotella, and Alistipes, which led to the remodeling of gut microbiota. In summary, RNPs significantly improved the conditions of mice with IBS-D by inhibiting the FoxO pathway and enhancing gut microbiota. This study concludes that RNPs could significantly improve the symptoms of mice with IBS-D through these means.

## 1. Introduction

One prevalent functional bowel illness is diarrheal irritable bowel syndrome (IBS-D) [1]. Patients with low inflammation in the intestinal mucosa are often accompanied by abdominal pain, distension, and diarrhea [2]. IBS is commonly divided into four subtypes, namely constipated IBS (IBS-C), diarrheal IBS (IBS-D), mixed bowel habits IBS (IBS-M), and unclassified IBS, based on the main fecal patterns that individuals report (IBS-U involves fecal types that incorrectly classify humans as one of the other three subtypes) [3], of which IBS-D is the most common subtype in the Rome IV standard [4].

It is thought that a variety of mechanisms, including visceral hypersensitivity, intestinal motility disorder, brain–gut axis disorder, a low degree of intestinal inflammation, genetics, social and psychological stress, and diet interact to cause the pathogenesis of IBS-D, which is still not fully understood [5]. Intensive investigations have shown that patients with this disease have a special visceral hypersensitivity state. It represents the increase in visceral sensitivity to stimulation, usually relying on the capacity threshold or the pressure threshold. Studies have shown that the initial sensory threshold and pain threshold of patients with irritable bowel syndrome during rectal balloon dilatation are significantly lower than those in the normal group, indicating that they may have increased visceral sensitivity [6]. Clinical studies have shown that PAR2-activated protease and PAR2 are potential common therapeutic targets for the treatment of abdominal pain in all patients with IBS [7].

*Ribes nigrum* L. is rich in nutrients, such as organic acids, vitamins, minerals, polyphenols [8], polysaccharides, and other bioactive substances. It has been discovered that polyphenols have numerous physiological actions, including those that are anti-inflammatory, antiviral, anti-tumor, blood lipid-lowering, and gut flora-regulating. Berry polyphenols have been widely used to treat diarrhea [9], ulcerative colitis [10], and IBS-D [11]. Studies have shown that polyphenols usually inhibit mitochondrial ATP synthesis to trigger AMPK activation, involving different downstream signaling cascades, such as the FoxO pathway [12]. Quercetin can reduce visceral sensitivity in PI-IBS rats [13], in addition, phytothymol can increase the intestinal transit time and decrease visceral sensitivity in rats with IBS [14].

There are more than 800 kinds of bacteria in human gut microbiota; the total is more than 10^14^, mainly including Bacteroides, Clostridium [15], Lactobacillus, Enterococcus, Escherichia coli, and Bifidobacterium [16,17]. Gut microbiota are involved in host metabolism, intestinal homeostasis maintenance, and several other important physiological processes. Many scientific studies have confirmed that IBS and gut microbiota are closely related. According to epidemiological research, the gut flora of patients with IBS differs from that of healthy individuals [18]. Compared with normal people, patients with IBS have overgrowth of intestinal bacteria and more facultative bacteria, such as Klebsiella and Enterococcus, while *E. coli*, lactic acid bacteria, and Bifidobacterium are significantly reduced [19]. Restoring normal gut microbiota can alleviate or eliminate symptoms associated with IBS. Therefore, gut microbiota disorder may be an important cause of IBS.

In this work, we investigated the effect of RNPs on IBS-D symptoms using an IBS-D mouse model and a network pharmacological approach. The findings demonstrate that RNPs could suppress the FoxO pathway, enhance gut microbiota, raise the number of helpful bacteria in the digestive tracts of mice with IBS-D, and lessen IBS-D symptoms. The goal of this research is to gather more empirical evidence supporting RNPs’ efficacy in treating IBS-D.

## 2. Materials and Methods

### 2.1. Materials and Reagents

*Ribes nigrum* L. were purchased from Bin County, Harbin City, Heilongjiang Province, China. Anhydrous ethanol was purchased from Yongda Chemical Reagent Co., Ltd. (Tianjin, China). Dialysis Tube MD25 (Mw 5000) was purchased from Biolebo Technology Co., Ltd. (Beijing, China). Rifaximin was purchased from Xinda Pharmaceutical Co., Ltd. (Zibo, China). Acetic acid was purchased from Fuyu Fine Chemical Co., Ltd. (Tianjin, China). Phenol red, formic acid, and methanol were purchased from Keyuan Biochemical Co., Ltd. (Heze, China). IL-4 ELISA kit, IL-6 ELISA kit, IL-10 ELISA kit, and TNF-α ELISA kit were purchased from Andy Huatai Biotechnology Co., Ltd. (Beijing, China). Hematoxylin and Eosin (alcohol soluble) were purchased from Huishi Biochemical Reagent Co., Ltd. (Shanghai, China). Paraffin was purchased from Huayong Paraffin Co., Ltd. (Shanghai, China). RNA Extraction Kit, reverse transcription kit and DNA extraction kit were purchased from Magen Biotechnology Co., Ltd. (Guangzhou, China). Tks Gflex DNA Polymerase was purchased from Takara Bio Inc. (Dalian, China).

### 2.2. Preparation of RNP Extract

The *Ribes nigrum* L. was freeze-dried and crushed into powder. The powder was placed in 65% ethanol solution, treated with 270 W ultrasonic power for 50 min, and then centrifuged to obtain the supernatant. The collected liquid was subjected to rotary evaporation to remove ethanol and dialyzed with a dialysis tube (5 k Da) [20]. The dialysate was concentrated by rotary evaporation and freeze-dried to obtain RNPs. The final extract was kept for examination at −80 °C in storage. The content of RNPs was 24.81 ± 0.52 mg GAE/g. The purity of RNPs was 64.8%.

### 2.3. UPLC-MS Analysis of Polyphenols in Ribes nigrum L.

Chromatographic conditions: Vanquish HPLC system and Q Exactive Focus mass spectrometry (LC-MS) were used to analyze the degradation products. Separation was performed on a Hypersil GOLD chromatographic column (100 × 2.1 mm, 1.9 μm, Thermo Fisher Scientific, Waltham, MA, USA), and the injection volume was 5 μL. The mobile phase was 0.1% formic acid (A) and methanol (B), and the following gradient elution (A/B, *v*/*v*) was used: 98: 2 in 0–1 min, 2: 98 in 9–12 min, and 98: 2 in 13–15 min. The flow rate was set to 0.35 mL/min [21,22].

Mass spectrometry conditions: Electrospray ion source (ESI) was used to scan and collect data in positive and negative ion modes. An MS analysis was performed using an electrospray ionization source in negative ion mode. The spray voltage was 3000 V, and the capillary temperature was 320 °C. MS acquisition was processed in full-scan mode in the range of 70 to 1000 Da with a mass resolution of 70,000.

Database and data processing: The chemical composition information of *Ribes nigrum* L. polyphenols was collected using the database included in the MS-DIAL 4.70 metabolomics analysis software, including the name of the compound and the ionization mode. The mass spectrometry fragment information was processed using Xcalibur4.1 software (Thermo Fisher Scientific) to obtain the final result.

Preparation of test solution: An amount of 1 g of freeze-dried powder of *Ribes nigrum* L. polyphenols was accurately weighed, and 25 mL of methanol was precisely added, and the mass was weighed. After ultrasonic treatment for 30 min, it was taken out and filtered through a 0.22 μm microporous membrane, and the filtrate was taken.

### 2.4. Network Pharmacological Analysis and Molecular Docking of IBS-D by RNPs

The Traditional Chinese Medicine Systems Pharmacology (TCMSP) Database (version 2.3, https://old.tcmsp-e.com (30 July 2024)) provided the RNP components. Potentially useful components were found by assessing the parameters related to absorption, distribution, metabolism, and excretion (oral bioavailability > 0.18; drug similarity > 30%). After that, information about the components that were found was integrated with data from RNPs and RNP mass spectrometry, and the TCMSP database provided information about their possible targets [23]. We entered the keyword “IBS-D” in the GenBank and DisGenet databases to search for targets associated with IBS-D. Screening was carried out for shared targets between RNPs and IBS-D. The putative RNP targets were entered into the DAVID database (https://david.ncifcrf.gov/ (7 August 2024)), and the KEGG pathway and biological function of GO were examined. The protein interaction network was established using the STRING database.

The selected RNP was docked with the key target, and the plane structure was drawn using ChemBio3D Ultra 14.0 software and transformed into a three-dimensional structure. The binding energy between the target and the compound was calculated, and molecular docking was carried out.

### 2.5. IBS-D Induction in Mice

Eight-week-old male KM mice were purchased from Pengyue Experimental Animal Breeding Co., Ltd. (Jinan, China) (Approval No. SCXK (lu) 2019 0003), and raised under SPF conditions (22 ± 2 °C, humidity of 40–60%, and 12 h light/dark cycle) in the School of Life Sciences, Northeast Forestry University. Sixty male mice in good health were split into the following groups at random: the model group (IBS group), the high-dosage RNP-H group (RNP-H group, 400 mg/kg/day), the low-dose RNP group (RNP-L group, 100 mg/kg/day), the medium-dose RNP group (RNP-M group, 250 mg/kg/day) [24], and the rifaximin group (RIF group) (Xinda Pharmaceutical Co., Ltd. (Zibo, China)). For the CON group, we provided the same volume of normal saline as RNPs once a day for 14 days. The mice in the IBS group were treated with 3% acetic acid solution enema combined with centrifuge tube restraint for 1 h per day for 7 days.

Under ether inhalation anesthesia, we injected 3% acetic acid (200 ± 20 μL) into the mouse colon at a depth of 3–4 cm from the anus and kept the mice head-down for 30 s to prevent leakage [25,26]. Then, we rinsed the mice with normal saline at a concentration of 200 ± 20 μL and suspended them until they woke up. This was repeated after 3 days. The model was made for 7 days, during which the mice were given 2 enemas with acetic acid and underwent 1 h of centrifugal tube restraint [27].

After the establishment of the model, the abdominal pain threshold was measured using the abdominal retracted reflex (AWR) test, and the mice with visceral hypersensitivity (water injection less than 0.1 mL) were identified as IBS-D models [28]. The Northeast Forestry University Ethics Committee for Experimental Animal Research accepted this work after considering the rights to biodiversity, clinical research, and animal experimentation.

### 2.6. Abdominal Withdrawal Reflex (AWR)

We performed colorectal distension stimulation on each group of mice and used the abdominal withdrawal reflex (AWR) [29] score to evaluate the visceral pain threshold. A score of 3 (abdominal muscle contraction during colon dilation and abdominal muscle lifting off the tabletop) was used as the visceral pain threshold to assess the visceral sensitivity of the mice [30], and the volume of injected water was recorded. Each mouse was measured three times, with a 5 min interval between each measurement, and the average of the three measurements was taken as the visceral pain threshold of the mice. The AWR scoring criteria are shown in Table 1.

### 2.7. Changes in Mice’s Weights and Feces in Every Group

We measured and recorded the body weight of the mice at the same time every day, fed each mouse separately, observed and recorded the shapes and defecation times of the feces, and then provided scores according to the Bristol feces table in Table 2. The feces of mice were collected within 2 h and baked in a constant temperature oven at 50 °C for 24 h, and then the dry weight of the feces was calculated. The weight changes in the feces before and after baking were recorded, and the moisture content of the feces was calculated.

(1) Fecal water content=(wet weight−dry weight)/wet weight×100%

We calculated the total number of feces and loose stools in mice within 6 h using the filter paper imprinting method. The judgment of loose stool is based on whether there are stains on the filter paper.

(2) $$Diluted\;stool\;rate=\frac{number\;of\;loose\;stool\;particles}{total\;number\;of\;stool\;particles}\times 100\%$$

### 2.8. Total Intestinal Transport Time

Before evaluation, all mice were fasted and allowed to drink water for 12 h. Oral injections of 0.2 mL of phenol red suspension were given to each animal. We recorded the time from gavage to the first round of red stool excretion in mice within 4 h.

### 2.9. Calculation of Visceral Index of Immune Organs

After fasting for 24 h on the last day, we weighed each group of mice. After euthanasia of mice by cervical dislocation, the thymus and spleen were taken and weighed after dehumidification with filter paper.

(3) Organ mass index=organ mass/body mass×100%

### 2.10. Serum Inflammatory Factor Level

After taking out the serum stored at −80 °C, we detected the levels of IL-4, IL-6, IL-10, and TNF-α in the serum samples strictly according to the instructions of the ELISA kit (Andy Huatai Biotechnology Co., Ltd. (Beijing, China)).

### 2.11. Histopathological Observation

The mice’s colonic tissue samples were 6 μm thick, and they were fixed in paraffin and stained with hematoxylin and eosin (H&E) so that their morphology and structure could be examined under a microscope.

### 2.12. Quantitative Reverse Transcription Polymerase Chain Reaction (Q-RTPCR)

At −20 °C, we isolated mouse colon RNA and reversed it to cDNA, performed Q-RTPCR, and repeated this process three times for each sample. The sequence of each gene primer is shown in Table 3 [31,32].

### 2.13. Fecal Microbiota Analysis

The sample’s genomic DNA was extracted using a DNA extraction kit (Magen Biotechnology Co., Ltd. (Guangzhou, China)), and agarose gel electrophoresis and Nano Drop2000 (Thermo Fisher Scientific (Waltham, MA, USA)) were used to measure the DNA’s concentration. Tks Gflex DNA Polymerase was utilized to carry out PCR using genomic DNA as a template, ensuring amplification precision and efficiency. The V3–V4 region of 16S rRNA was selected for gene amplification and sequencing. The primer sequences were 343F (5′-TACGGRAGGCAGCAG-3′) and 798R (5′-AGGGTATCTAATCCT-3′) [33]. The PCR products were identified using electrophoresis, isolated using magnetic beads, and then utilized as a template for additional PCR amplification, purific ation, and Qubit quantification. The same volume of sample was combined and sequenced based on the PCR product’s concentration.

### 2.14. Statistical Analysis

The statistical software SPSS 23.0 was utilized for data analysis, and the experimental data are presented as the mean ± standard deviation (SD). To compare groups, a statistical analysis was performed using an analysis of variance (ANOVA) followed by Tukey’s test. Significant differences were indicated by * *p* < 0.05 and ** *p* < 0.01.

## 3. Results

### 3.1. Analysis of RNPs by UPLC-MS

**Figure 1:**
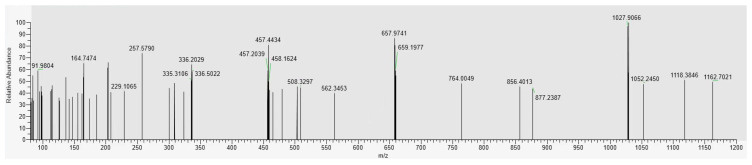
TIC MS diagram of RNP in positive mode.

The components of the RNPs were analyzed using UPLC-MS. The total ion current diagram of the RNPs was obtained (Figure 1). Combined with the molecular weight of each compound (Table 4), the components in the RNPs were identified as phloretin, thymol, sinapic acid, quercetin, coumaric acid, luteolin, etc.

### 3.2. Network Pharmacology Analysis of Effect of RNPs on IBS-D

**Figure 2:**
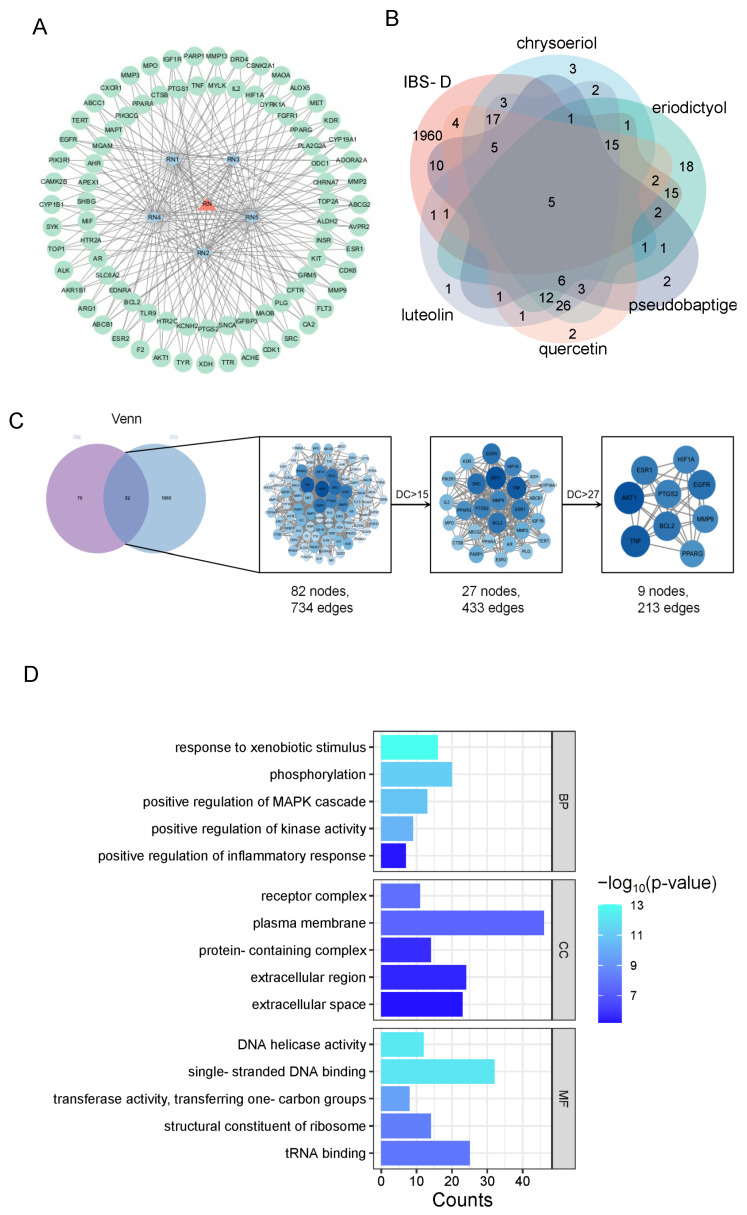

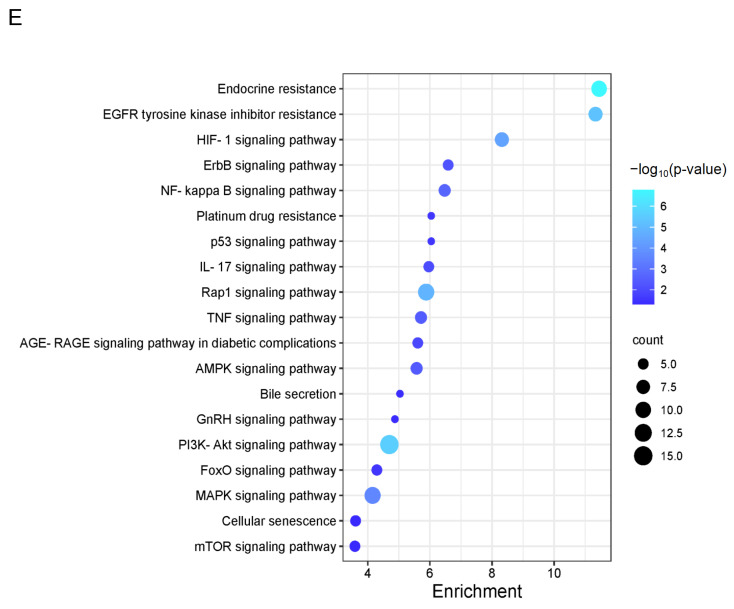
Network pharmacological analysis of RNP ingredients and IBS-D. (**A**) Target network of active component. (**B**) Identification of possible RNP treatment targets. (**C**) Intersection of core target screening and possible RNP component targets with IBS-D illness targets. (**D**) Analysis of GO Biological Function Enrichment. (**E**) Target enrichment of KEGG pathways using chemicals that cross paths.

By analyzing the components of RNPs using UPLC-MS and a network pharmacology analysis, five polyphenolic compounds were screened out from the RNPs: pseudobaptigenin, eriodictyol, chrysoeriol, quercetin, and luteolin. We constructed an active ingredient target network using Cytoscape, as shown in Figure 2A. Figure 2B shows possible targets for RNP therapy. We collected and crossed targets of RNP components and IBS-D and obtained 82 common targets. Afterwards, a protein–protein interaction network was constructed using Cytoscape 3.9.1 software, as shown in Figure 2C. Nine core protein targets, including AKT1, TNF, EGFR, and BCL2, were screened based on degree, closeness, and betweenness indicators. Among them, AKT1 protein, also known as PKB, is a key component in the cell signaling pathway, as it is involved in regulating various processes such as cell growth, survival, metabolism, and differentiation. According to research, AKT is a key regulatory factor in the FoxO pathway [34]; FoxO transcription factors are phosphorylated/threonine protein kinase B (PKB)/Akt downstream targets [35]. TNF refers to tumor necrosis factor, which is a cytokine naturally produced by macrophages in response to a bacterial infection or other immunogenic reactions. It works in synergy with interferon to kill tumor cells. EGFR is a receptor for EGF cell proliferation and signaling. Research has shown that EGFR is involved in signal pathway dysregulation in cancer cells, typically occurring in colorectal cancer cells [36,37]. The BCL2 family is a group of proteins closely related to the regulation of cell apoptosis, mainly playing a role in the mitochondrial membrane of cells. The decrease in their activity can lead to excessive cell proliferation and inflammation [38].

Figure 2D shows the target Gene Ontology functional enrichment results of the effects of RNPs on IBS-D. A Gene Ontology functional enrichment analysis is divided into the Biological Process (BP), Cellular Component (CC), and Molecular Function (MF). Positive control of the inflammatory response, receptor complex, plasma membrane, DNA helicase activity, and single-stranded DNA binding are among these targets. The following are the KEGG pathway enrichment results that are displayed in Figure 2E: the pathways associated with FoxO, PI3K-Akt, AMPK, mTOR, TNF, HIF-1, EGFR tyrosine kinase inhibitor resistance, and MAPK signaling.

### 3.3. Molecular Docking of RNP with Core Targets

**Figure 3:**
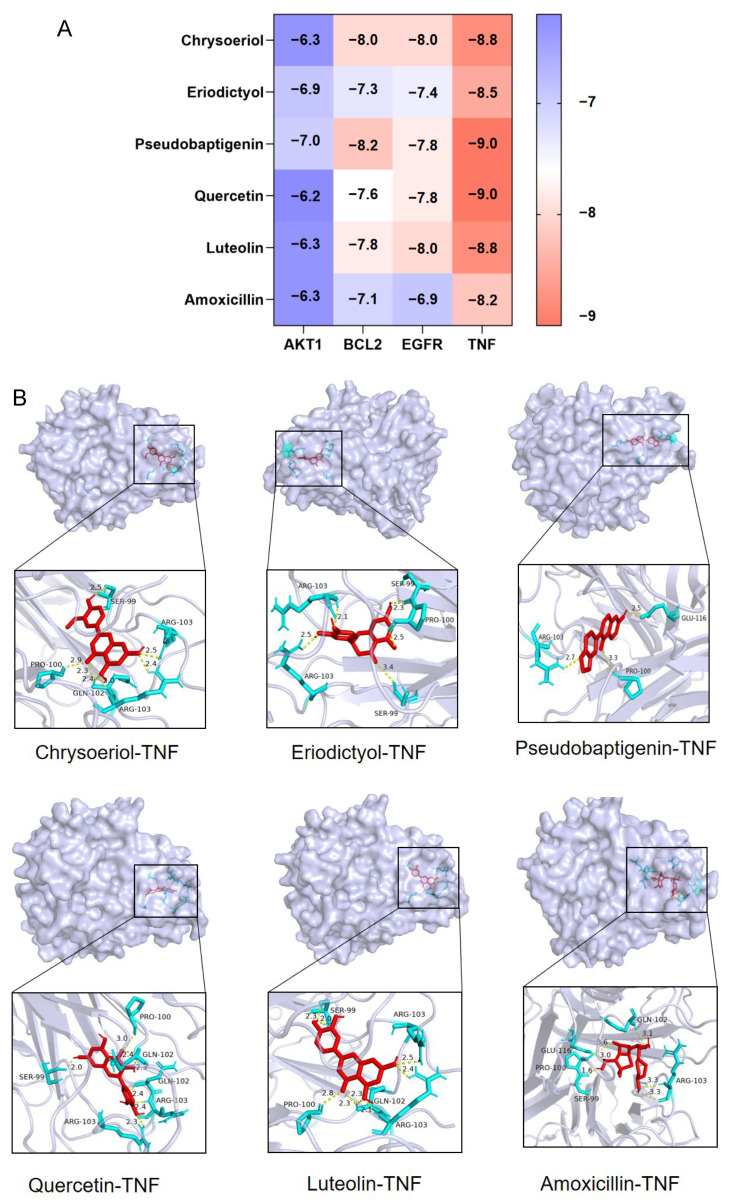
Molecular docking results of RNP and core targets. (**A**) Molecular docking results of active ingredient and key targets. (**B**) Macromolecular docking diagram of active ingredient and key target.

By combining a UPLC-MS analysis of polyphenols in black currant and screening of the TCMSP database, we selected five main active components of RNPs for molecular docking, and amoxicillin, the main component of rifaximin, was used as a positive control. The top four core targets, AKT1, TNF, EGFR, and BCL2, were ranked by degree. To further investigate potential binding interactions, a molecular docking analysis was conducted to evaluate the binding affinity of these components to their corresponding targets. The lower the molecular docking fraction (kcal/mol) value, the greater the likelihood of binding between two compounds. Usually, a binding energy < 0 indicates spontaneous binding, while a binding energy <−5 indicates strong binding affinity. In this study, all binding energies were below −6.0 kcal/mol, as shown in Figure 3A. This indicates that the five main components of RNPs have strong binding activity with key targets of AKT1, TNF, EGFR, and BCL2. Figure 3B shows the molecular docking results of four core targets and active ingredients.

### 3.4. RNP Alleviates Diarrhea in Mice with IBS-D

**Figure 4:**
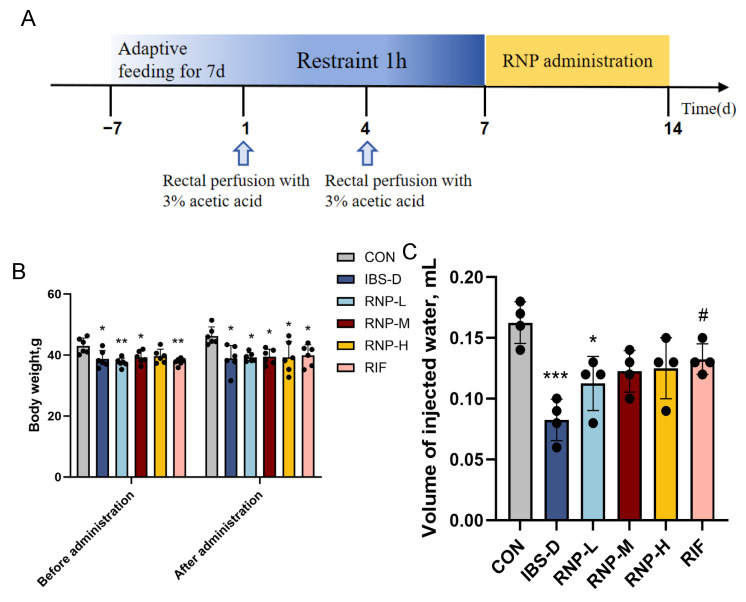

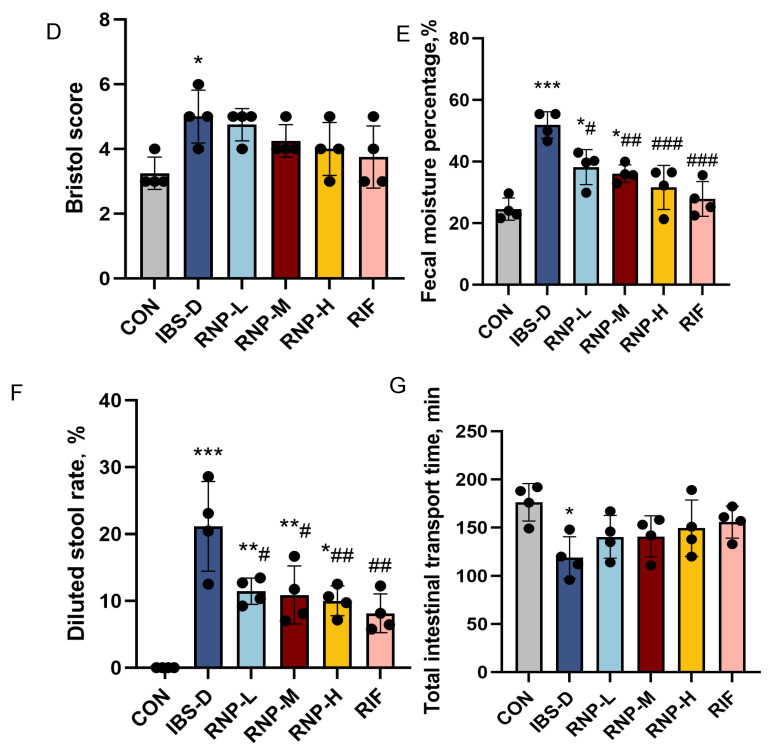
RNPs can improve the intestinal symptoms of mice with IBS-D. (**A**) A schematic diagram of the RNP administration pipeline in mice with IBS-D. (**B**) The body weights. (**C**) The volume of injected water. (**D**) The Bristol score. (**E**) The fecal moisture percentage. (**F**) The diluted stool rate. (**G**) The total intestinal transport time. *n* = 4–6 tests/group. The mean ± SD is used to express values. All statistical comparisons with the CON group are indicated with the symbols * *p* < 0.05, ** *p* < 0.01 and *** *p*< 0.001. Comparisons with the IBS-D group are denoted as # *p* < 0.05, ## *p* < 0.01 and ### *p*< 0.001.

In order to determine the improvement effect of RNPs on IBS-D, we established an IBS-D mouse model (Figure 4A). Figure 4B shows that both before and after receiving RNP treatment, the body weights of the mice treated with IBS-D were noticeably lower than those of the control animals (*p* < 0.05). The visceral sensitivity of the mice was measured using the volume of injected water. From Figure 4C, it can be seen that compared with the CON group, the water injection volume of the mice in the IBS group decreased significantly (*p* < 0.01), indicating that the mice in the IBS group were the most sensitive to pain. The water injection volume of the mice treated with RNP and RIF was improved, indicating that RNPs and RIF can alleviate visceral hyperalgesia. By observing the feces of mice in each group, it was found that the feces of mice in the IBS-D group were softer, which can be reflected by the increase in the Bristol score (Figure 4D). After calculating the water content in the feces of the mice in each group, it was discovered that the IBS-D group had a considerably higher water content in their feces (*p* < 0.01), which was significantly improved after RNP treatment (Figure 4E). According to Figure 4F, after receiving RNP treatment, the rate of loose stool in the IBS-D group dramatically decreased (*p* < 0.05) and was significantly higher (*p* < 0.01) than that in the CON group. The total intestinal transit time can reflect the intestinal movements of mice, which can be seen in Figure 4G. When compared to the CON group, the IBS group’s total intestinal transit time was significantly shorter; however, no discernible change was observed when compared to the RNP and RIF groups (*p* > 0.05).

### 3.5. Effect of RNPs on Immune Index of Mice in Each Group

**Figure 5:**
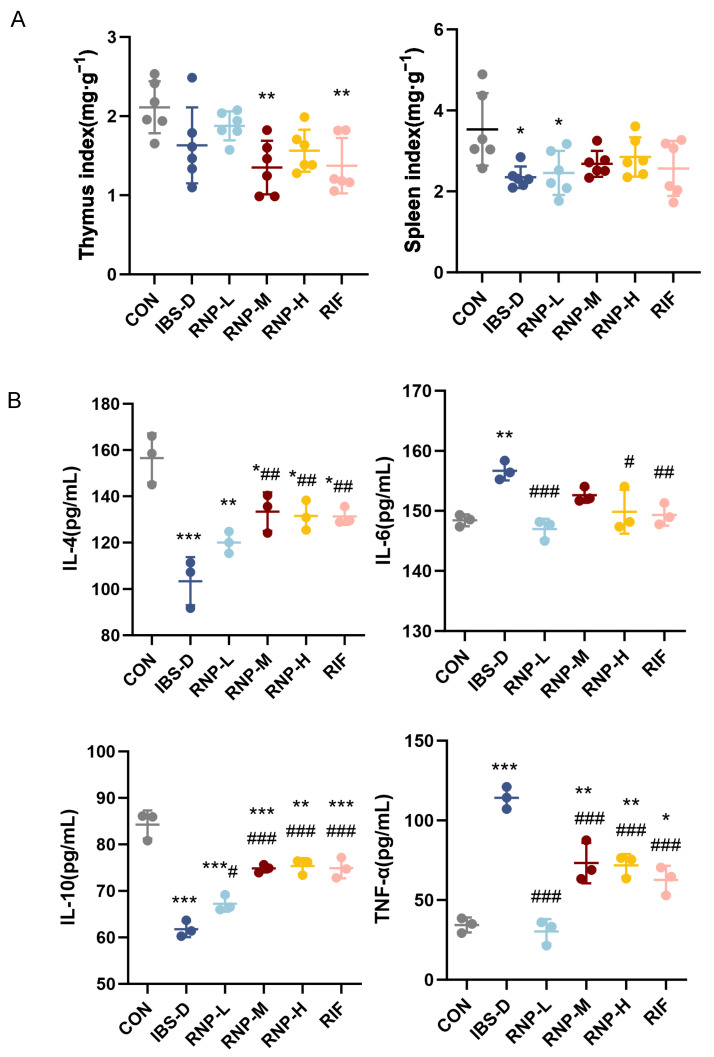
Effects of RNP on immune index and inflammatory factors in mice. (**A**) Thymus index and spleen index. (**B**) Inflammatory factor. *n* = 3–6 tests/group. Mean ± SD is used to express values. All statistical comparisons with the CON group are indicated with the symbols * *p* < 0.05, ** *p* < 0.01 and *** *p* < 0.001. Comparisons with the IBS-D group are denoted as # *p* < 0.05, ## *p* < 0.01 and ### *p* < 0.001.

According to Figure 5A, the spleen index of the mice with IBS-D was significantly lower than that of the CON mice (*p* < 0.05), indicating that the thymus of the mice with IBS-D underwent atrophy.

### 3.6. The Effect of RNPs on the Contents of Inflammatory Factors in Mouse Serum

Both IL-4 and IL-10 are significant anti-inflammatory cytokines that are essential for anti-inflammatory actions in the inflammatory response. Pro-inflammatory cytokines like TNF-α and IL-6 have various functions in the immune and inflammatory systems. Figure 5B illustrates that the IBS group had considerably lower levels of IL-4 and IL-10 (*p* < 0.01) in comparison to the CON group, although there was a significant rise (*p* < 0.01) in IL-6 and TNF-α levels. Furthermore, in the IBS-induced animals, RNPs and RIF can raise IL-4 and IL-10 levels (*p* < 0.01) while lowering TNF-α and IL-6 levels (*p* < 0.05).

### 3.7. H&E Staining of Colonic Mucosal Tissue

**Figure 6:**
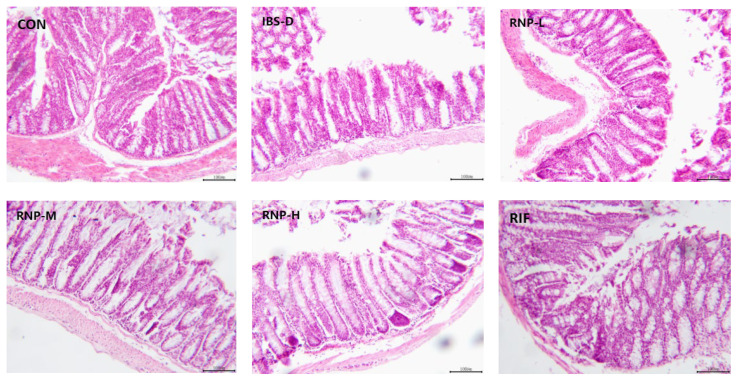
H&E staining of colon.

It can be seen from Figure 6 that the colon mucosa in the CON group is arranged neatly and tightly with a clear cell structure and a large number of goblet cells. No edema, vascular congestion, or inflammatory infiltration was observed. The mucosal tissue structure of the IBS group was looser and more disorganized than that of the CON group and had a significant proliferation of connective tissue, cell atrophy, unclear cytoplasmic and nuclear boundaries, a significantly reduced number of goblet cells, local tissue edema, and inflammatory infiltration in the submucosal layer. Compared with the IBS group, the treatment group showed some improvement in the pathological manifestations mentioned above, with the RNP-H group and RIF group showing the most significant improvement.

### 3.8. In Mice with IBS-D, BSD Controls the Expression of FoxO1, FoxO3a, and FoxO4

**Figure 7:**
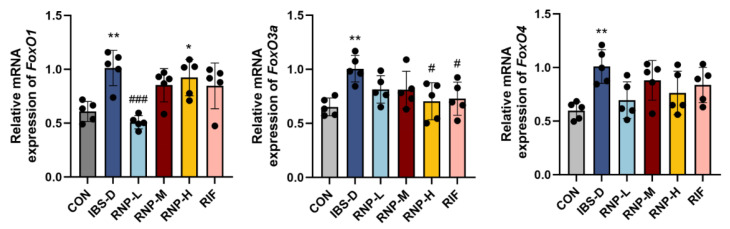
The mRNA levels of FoxO1, FoxO3a, and FoxO4. *n* = 5 tests/group. The mean ± SD is used to express values. All statistical comparisons with the control group are indicated with the symbols * *p* < 0.05 and ** *p* < 0.01. Comparisons with the IBS-D group are denoted as # *p* < 0.05 and ### *p*< 0.001.

Through a previous network pharmacology analysis, the KEGG pathway was enriched to the FoxO pathway. Thus, we looked at the four FoxO signaling pathway components’ mRNA levels. Figure 7 illustrates the considerable increase (*p* < 0.01) in the mRNA levels of FoxO1, FoxO3, and FoxO4 in the intestines of mice with IBS-D compared to the CON group. Furthermore, the colons of mice treated with RNPs and RIF had significantly lower levels of FoxO1 and FoxO3a mRNA expression than the colons of mice in the IBS-D group (*p* < 0.05). Furthermore, there was a significant difference (*p* < 0.01) in the amount of FoxO1 mRNA in the colons of mice in the the RNP-L and IBS-D groups.

### 3.9. In Mice with IBS-D, RNP Therapy Modifies the Intestinal Microbiome

**Figure 8:**
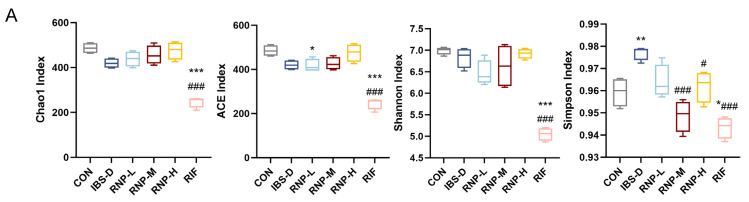

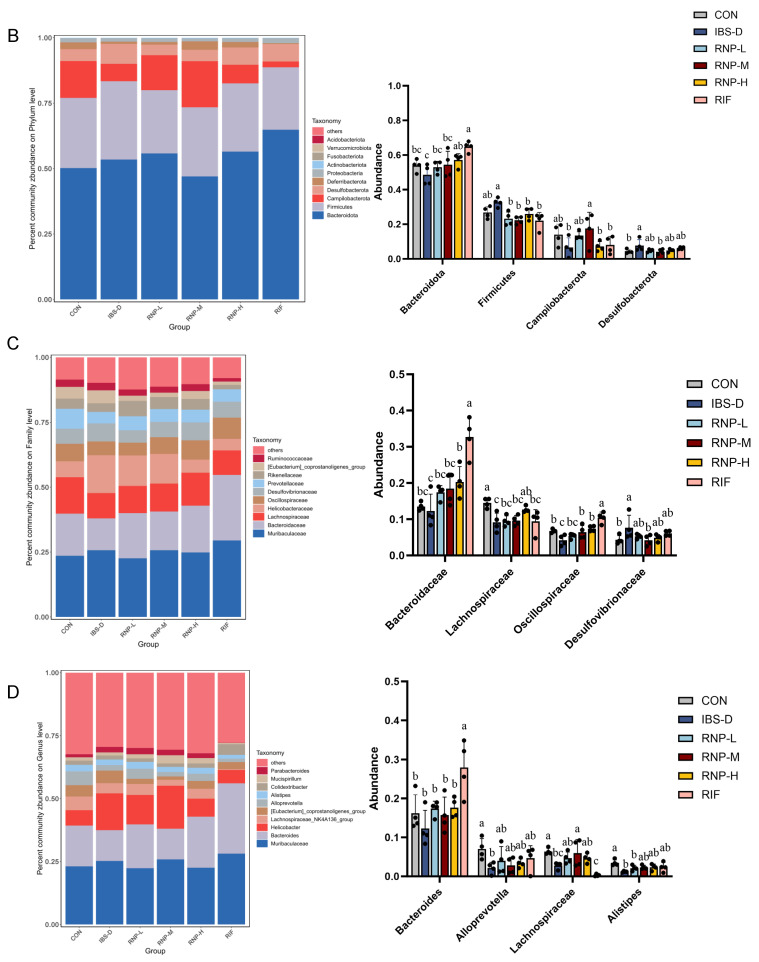
RNP treatment remodels gut microbiota in mice with IBS-D. (**A**) Alpha diversity analysis for every group. (**B**) Analysis based on phylum. (**C**) Analysis at family level. (**D**) Analysis at genus level. *n* = 4 tests/group. Mean ± SD is used to express values. All statistical comparisons with the CON group are indicated with the symbols * *p* < 0.05, ** *p* < 0.01 and *** *p* < 0.001. Comparisons with the IBS-D group are denoted as # *p* < 0.05 and ### *p* < 0.001.

In order to determine the effect of RNPs on the gut microbiota structure of mice with IBS-D, the Shannon index analysis, Simpson index, Chao1 index, and Ace index were used to analyze the richness and diversity of the gut microbiota in mice. There was a significant difference compared with the CON group (*p* < 0.05). Furthermore, after RNP treatment, these indices showed varying degrees of reversal (*p* < 0.05) (Figure 8A). In the observed changes in the gut microbiota composition, studies at the phylum level revealed decreases in Bacteroidota and Campilobacterota in the IBS group, while the Firmicutes and Desulfobacterota significantly increased (Figure 8B). Firmicutes and Bacteroidetes can generate intestinal free radical scavengers and anti-inflammatory factors [39], thereby inhibiting intestinal oxidative stress and inflammatory responses. In addition, the increase in the ratio of Firmicutes to Bacteroidetes also reduces intestinal immune activity [40]. It can be seen in Figure 8C that at the family level, it was found that compared with the CON group, the concentrations of Bacteroidaceae, Lachnospiraceae, and Oscillospiraceae in the intestinal tracts of the mice in the IBS group significantly decreased (*p* < 0.05), while they significantly increased after RNP and RIF treatment, especially in the RIF group, where Bacteroidaceae increased the most. Among them, Lachnospiraceae is involved in the synthesis of SCFA, and its abundance is associated with gastrointestinal diseases such as IBD [41]. Figure 8D shows that at the genus level, concentrations of Bacteroides, AlloPrevotella, Lachnospiraceae, and Alistipes in the IBS group are all lower than those in the CON group (*p* < 0.05). Among them, the reduction in Alistipes will increase intestinal permeability, leading to intestinal inflammation. In summary, RNPs improved gut microbiota diversity in mice with IBS-D.

## 4. Discussion

A persistent functional gastrointestinal disorder, IBS, affects 5–10% of people worldwide. IBS is becoming more common every year and affects people of all ages due to the accelerated pace of life and rising work pressure. IBS seriously affects patients’ work, study, lives, and mental health and reduces the quality of life of patients. Research has indicated that individuals with IBS-D frequently exhibit mental and psychological disorders, and there is a strong correlation between the mental health of these patients and their quality of life [42,43]. However, there is still a lack of effective treatment, and patients often visit doctors repeatedly, causing huge economic and psychological burden. The etiology and pathogenesis of IBS have not yet been fully clarified. At present, studies have found that reducing the content of 5-HT can alleviate the symptoms of IBS [44]. Aminosalicylic acid can also improve the overall symptoms of patients with diarrhea-predominant IBS, but the quality of evidence is low [45]. Studies have also shown that dietary intervention can be used as the main treatment for IBS symptoms [46]. Researchers recommend a normal and healthy diet as the preferred diet therapy for patients with non-constipation IBS [47]. IBS-D has a complicated etiology, and there is currently no practical or efficient cure. Therefore, finding more effective and safer treatment methods is the focus of IBS-D research.

The complex pathogenesis of IBS mainly includes changes in visceral sensitivity, low-grade inflammation of the intestinal mucosa, changes in intestinal microecology, and psychosocial factors. Increased sensitivity or pain perception of the body’s internal organs is referred to as visceral hypersensitivity. Visceral allergy is considered to be the core pathogenesis of IBS-D. There are also more methods for the research on and preparation of animal models of IBS-D, mainly including restraint stress stimulation, senna gavage, physical and chemical factor stimulation, maternal and infant separation, gene knockout, and a variety of combined stimulation methods. The acetic acid stimulation method is widely used in the study of IBS-D models because of its simple operation and short molding cycle. Considering that psychological stress and other factors are high risk factors for IBS-D [48], and there have been instances of acetic acid mixed with restraint stress, to create the mouse model of IBS-D, we combined 3% acetic acid enema with centrifuge tube restraint using visceral sensitivity as the benchmark for successful modeling.

Plant polyphenols are a class of secondary metabolites with a phenolic structure which are widely distributed in fruits, roots, skins, leaves, and other tissues and organs of plants. They perform a wide range of biological tasks, including regulating gut flora, preventing obesity, and combating inflammation and free radical damage. *Ribes nigrum* L. is rich in polyphenols. In this study, polyphenols were extracted from *Ribes nigrum* L. by ethanol extraction and the ultrasound-assisted method, and combined with membrane technology dialysis, low-molecular-weight phenolic compounds could be retained, and high-molecular-weight carbohydrates could be removed. We found several compounds of *Ribes nigrum* L. polyphenols using UPLC-MS, such as pseudobaptigenin, eriodictyol, chrysoeriol, quercetin, and luteolin. Among them, in the process of the biotransformation of quercetin, hydroxylation, methylation, and deglycosylation reactions occur [49,50], and quercetin has been shown to induce TFEB-mediated lysosomal activation and then promote ferritin degradation, leading to ferroptosis and Bid-involved apoptosis [51]. Studies have shown that eriodictyol can alleviate ALI-related oxidative stress and apoptosis by activating the PI3K/AKT signaling pathway [52]. There is evidence that luteolin is an anti-aging active ingredient that improves aging by altering the interaction between p16 and CDK6 [53]. Research indicates that baizhu shaoyao decoction high in polyphenols can enhance intestinal permeability, brain–gut peptide levels, and depressive behavior caused by IBS-D by boosting the number of intestinal tight junctions [54]. Through CSF2-mediated NF-κB and JAK-STAT pathways, polyphenols contained in navel orange peel have been shown to reduce intestinal inflammatory damage and protect intestinal barrier integrity [55]. These studies have shown that polyphenols have certain therapeutic potential for IBS-D. However, it has been reported that the total phenolic content of the phenolic extract of cherry pomace decreased significantly during in vitro digestion. Due to the increase in pH in the intestine, the phenolic substances of large molecules will hydrolyze to form small molecules, and the total phenolic content will decrease. In addition, the reason why bound phenols are more degraded in the stomach may be the effect of digestive enzymes and bile acids on food during digestion, which can release bound phenols [56]. Therefore, in order to explore the effect of RNPs on IBS-D, after 7 days of modeling, RNP or RIF intervention was started for 14 days. We found that the body weights of the IBS-D-induced mice was significantly lower than that of the control mice. Serum inflammatory factors (IL-4, IL-6, IL-10, and TNF-α) were elevated, intestinal transport was accelerated, visceral sensitivity was increased, and the loose stool rates, fecal Bristol scores, and fecal water contents of the mice with IBS-D were all significantly higher. These findings are in line with the traits of IBS-D. Abdominal pain and all symptoms associated with diarrhea were significantly reduced by both RNP therapy and RIF therapy. These results demonstrate RNPs’ remarkable effectiveness in treating mice with IBS-D.

We enriched the FoxO pathway through a network pharmacology analysis, and the core targets such as AKT1 and EGFR were also closely related to the FoxO pathway. Serine/threonine protein kinase B (PKB)/Akt directs FoxO transcription factors downstream. The processes of cellular survival and proliferation are regulated by the Akt kinase [35]. Aberrant EGFR activity in COPD airways enhances the PI 3-kinase/Akt-mediated phosphorylation of FoxO3a, which reduces nuclear FoxO3a and increases the production of chemokines [37]. Through controlling gene expression, the FoxO transcription factor family affects a variety of physiological processes in cells, such as apoptosis, cell cycle regulation, glucose metabolism, resistance to oxidative stress, and lifetime extension. Studies have shown that green tea polyphenols may activate FoxO1 through Akt and AMPK to regulate ET-1 promoter [57]. Salvianolic acid B has been shown to inhibit the expression of acetylated FOXO1 through the up-regulation of SlRT3 [58]. Otherwise, the BSD-induced remission of IBS-D is facilitated by the FoxO signaling pathway. Additionally, FoxO 1 inhibitors significantly reduced the symptoms of IBS-D in mice, but FoxO 3a agonists had the opposite effect [54]. To find the mRNA levels of associated FoxO pathway genes, we employed Q-RTPCR. The findings demonstrate that the intestinal mRNA levels of FoxO1, FoxO3a, and FoxO4 in the mice with IBS-D were notably greater than those in the control mice. IBS-D and the FoxO signaling pathway may be related.

The intestinal tracts of mice are large and complex micro-ecosystems like those of humans. Under healthy conditions, a certain proportion of each genus is maintained to maintain the stability of the intestinal micro-ecosystem. Normal gut microbiota act as a body barrier to resist the invasion of foreign pathogenic bacteria. But once the pathogenic factors upset the equilibrium of intestinal microecology, the growth and procreation of pathogenic bacteria cause an imbalance in the gut microbiota, which is reflected in the increasing ratio of harmful pathogenic bacteria, like Fusobacterium and Streptococcus, and the decreasing ratio of probiotics like Lactobacillus. This causes diarrhea and visceral hypersensitivity. According to studies, dietary polyphenols and their metabolites perform a prebiotic role in promoting the growth of helpful bacteria and preventing the growth of harmful bacteria, which helps to maintain intestinal health. They also manage the balance of intestinal microbes. Colonic bacteria may transform polyphenols into bioactive substances that alter the gut ecology and impact host health [59]. According to studies, oral polyphenols can lessen intestinal inflammation and oxidative stress in the brain by enhancing the gut microbiota of mice that are fed a high-fat diet. Coffee cherry husks reduce brain inflammation in colitis-affected rats via controlling the gut microbiota and blocking the NF-κB signaling pathway [60]. Mice with IBS-D were treated with RNPs. First, we observed significant changes in the gut microbiota of mice with IBS-D and control mice. The Chao1 index, ACE index, and Shannon index of the mice with IBS-D were significantly lower than those of the control mice, while the Simpson index was significantly higher than that of the control mice. These indexes indicate that the richness and diversity of gut microbiota in the mice with IBS-D were significantly lower than those in the control mice. Our results show that RNP could improve the damaged gut microbiota of mice with IBS-D. In mice with IBS-D, RNPs may augment the Bacteroidota population while diminishing the Firmicutes at the phylum level. And with the increase in RNP dose, the content of Bacteroidota also increased. In the mice with IBS-D, there was a decrease in the relative abundances of Bacteroidaceae, Lachnospiraceae, and Oscillospiraceae families. Of these, Lachnospiraceae is a potentially helpful bacteria that is found in the intestines of the majority of healthy individuals [61]. At the genus level, we found that the relative abundance of Helicobacter in mice with IBS-D was significantly increased, while Helicobacter caused chronic gastritis and led to gastric ulcer and atrophy, and in severe cases, gastric cancer in the control mice. After treatment with RNPs or RIF, the relative abundance of Helicobacter decreased.

## Figures and Tables

**Table 1:** AWR scoring criteria.

Score	Standard for Evaluation
One	When the colon dilates, the body stays still and the head moves less.
Two	When the colon dilates, the abdominal museles contract but do not lift off the table.
Three	When the colon dilates, the abdominal muscles contract and lift off the table.
Four	When the colon expands, the pelvis is raised, the body is arched, and the perineum is lifted off the ground.

**Table 2:** Bristol stool chart.

Score	Character
1. Pyreniform	Thick and rough, akin to sheep’s stool
2. Dry hard shape	Surface is convex and texture is firm
3. There are folds	Banana-shaped, with surface that is wrinkled
4. Banana shape	Banana-shaped, flawless exterior
5. Soft poop	Texture is semi-solid and supple
6. Porridge like	Unchangeable form, atherosclerosis
7. Water like	No samples of solids or water

**Table 3:** Sequences of primers for each gene.

Gene	Sequence
FOXO3a(F)	CATGTAGAGTGTTGTGGAGAGC
FOXO3a(R)	AACGGCTGGCCTGTCCTGAA
FOXO1(F)	CGTCCTCGAACCAGCTCAA
FOXO1(R)	TTGGCGGTGCAAATGAATAG
FOXO4(F)	GGTGCCCTACTTCAAGGACAA
FOXO4(R)	ATCGGGGTTCAGCATCCA

**Table 4:** Composition of polyphenol compounds in *Ribes nigrum* L.

Component	Retention Time (min)	Compound	MS (*m*/*z*)	Chemical Formula
1	0.991	Citric acid	215.0147	C_6_H_8_O_7_
2	2.950	Nardosinone	251.1599	C_15_H_22_O_3_
3	1.385	Phloretin	275.108	C_15_H_14_O_5_
4	4.193	Eriodictyol-7-O-glucoside	449.1049	C_21_H_22_O_11_
5	7.111	Thymol	149.0949	C_10_H_14_O
6	3.067	Sinapic acid	223.062	C_11_H_12_O_5_
7	6.182	Tectorigenin	299.1079	C_16_H_12_O_6_
8	4.051	Quercetin-3-O-glucoside	505.0917	C_21_H_20_O_12_
9	4.051	Hypericin	505.0917	C_30_H_16_O_8_
10	0.936	Fumaric acid	139.0014	C_4_H_4_O_4_
11	4.820	Quercetin	303.0481	C_15_H_10_O_7_
12	13.689	Aconitic acid	196.9994	C_6_H_6_O_6_
13	6.182	Coumaric acid	163.0375	C_9_H_8_O_3_
14	1.185	2-Hydroxychalcone	223.129	C_15_H_12_O_2_
15	0.991	Luteolin	286.236	C_15_H_10_O_6_
16	3.270	Pseudobaptigenin	281.0492	C_16_H_10_O_5_
17	4.993	Eriodictyol	287.0532	C_15_H_12_O_6_
18	4.736	Kaempferol-4′-glucoside	471.0877	C_21_H_20_O_11_
19	1.018	Rosmarinic acid	399.0479	C_18_H_16_O_8_
20	14.732	Chrysoeriol	299.0603	C_16_H_12_O_6_
21	4.680	Coumaroyl quinic acid	337.0868	C_16_H_18_O_8_
22	1.0179	Caffeoyl quinic acid	353.0815	C_16_H_18_O_9_
23	13.718	Veratric acid	180.9888	C_9_H_10_O_4_
24	3.532	Catechin	289.124	C_15_H_14_O_6_
25	6.442	3-Hydroxyflavone	237.1086	C_15_H_10_O_3_
26	5.135	Magnolol	311.125	C_18_H_18_O_2_
27	6.182	Dihydroresveratrol	229.0818	C_14_H_14_O_3_
28	5.050	Isorhamnetin 3-galactoside	501.097	C_22_H_22_O_12_
29	4.109	3-O-Feruloylquinic acid	391.1921	C_17_H_20_O_9_
30	4.250	Chlorogenic acid	377.0818	C_16_H_18_O_9_
31	3.851	Cyanidin-3-glucoside chloride	484.8	C_21_H_21_ClO_11_
32	4.193	Kaempferol-3-O-pentoside	417.0757	C_20_H_18_O_10_
33	14.092	Myricetin-3-O-xyloside	449.071	C_20_H_18_O_12_

## Data Availability

The original contributions presented in the study are included in the article, further inquiries can be directed to the corresponding author.

## Abbreviations

AKT1, serine/threonine-protein kinase; AMPK, adenosine 5′-monophosphate (AMP)-activated protein kinase; AWR, abdominal withdrawal reflex; BCL2, B-cell lymphoma-2; BP, biological process; CC, cellular component; FoxO, forkhead box O; EGFR, epidermal growth factor receptor; H&E, hematoxylin–eosin; HIF-1, hypoxia inducible factor-1; UPLC-MS, ultra performance liquid chromatography–tandem mass spectrometry; IBS, irritable bowel syndrome; IBS-D, diarrheal irritable bowel syndrome; IL-4, interleukin-4; IL-6, interleukin-6; IL-10, interleukin-10; MAPK, mitogen-activated protein kinase; MF, molecular function; mTOR, mammalian target of rapamycin; PI3K-Akt, phosphatidylinositol 3-kinase/protein Kinase B; q-RTPCR, quantitative reverse transcription polymerase chain reaction; RNPs, *Ribes nigrum* L. polyphenols; TCMSP, Traditional Chinese Medicine Systems Pharmacology; TNF, tumor necrosis factor; TNF-α, tumor necrosis factor-α.

## References

[1] Lacy B.E., Mearin F., Chang L., Chey W.D., Lembo A.J., Simren M., Spiller R. (2016). Bowel disorders. Gastroenterology.

[2] Camilleri M. (2021). Diagnosis and treatment of irritable bowel syndrome: A review. JAMA.

[3] Longstreth G.F., Thompson W.G., Chey W.D., Houghton L.A., Mearin F., Spiller R.C. (2006). Functional bowel disorders. Gastroenterology.

[4] Oka P., Parr H., Barberio B., Black C.J., Savarino E.V., Ford A.C. (2020). Global prevalence of irritable bowel syndrome according to Rome III or IV criteria: A systematic review and meta-analysis. Lancet. Gastroenterol. Hepatol..

[5] Munjal A., Dedania B., Cash B. (2019). Update on Pharmacotherapy for Irritable Bowel Syndrome. Curr. Gastroenterol. Rep..

[6] Posserud I., Syrous A., Lindström L., Tack J., Abrahamsson H., Simrén M. (2007). Altered rectal perception in irritable bowel syndrome is associated with symptom severity. Gastroenterology.

[7] Cenac N., Andrews C.N., Holzhausen M., Chapman K., Cottrell G., Andrade-Gordon P., Steinhoff M., Barbara G., Beck P., Bunnett N.W. (2007). Role for protease activity in visceral pain in irritable bowel syndrome. J. Clin. Investig..

[8] Butnariu M. (2014). Detection of the polyphenolic components in *Ribes nigrum* L.. Ann. Agric. Environ. Med..

[9] Candellone A., Cerquetella M., Girolami F., Badino P., Odore R. (2020). Acute diarrhea in dogs: Current management and potential role of dietary polyphenols supplementation. Antioxidants.

[10] Li F., Yan H., Jiang L., Zhao J., Lei X., Ming J. (2021). Cherry polyphenol extract ameliorated dextran sodium sulfate-induced ulcerative colitis in mice by suppressing wnt/β-catenin signaling pathway. Foods.

[11] Chiarioni G., Popa S.L., Ismaiel A., Pop C., Dumitrascu D.I., Brata V.D., Duse T.A., Incze V., Surdea-Blaga T. (2023). The effect of polyphenols, minerals, fibers, and fruits on irritable bowel syndrome: A systematic review. Nutrients.

[12] Chu A.J. (2022). Quarter-century explorations of bioactive polyphenols: Diverse health benefits. Front. Biosci. Landmark.

[13] Qin H.-Y., Zang K.-H., Zuo X., Wu X.-A., Bian Z.-X. (2019). Quercetin attenuates visceral hypersensitivity and 5-hydroxytryptamine availability in postinflammatory irritable bowel syndrome rats: Role of enterochromaffin cells in the colon. J. Med. Food.

[14] Subramaniyam S., Yang S., Diallo B.N.t., Fanshu X., Lei L., Li C., Tastan Bishop Ö., Bhattacharyya S. (2020). Oral Phyto-thymol ameliorates the stress induced IBS symptoms. Sci. Rep..

[15] Atarashi K., Tanoue T., Shima T., Imaoka A., Kuwahara T., Momose Y., Cheng G., Yamasaki S., Saito T., Ohba Y. (2011). Induction of colonic regulatory T cells by indigenous Clostridium species. Science.

[16] Sekirov I., Russell S.L., Antunes L.C.M., Finlay B.B. (2010). Gut microbiota in health and disease. Physiol. Rev..

[17] Rivière A., Selak M., Lantin D., Leroy F., De Vuyst L. (2016). Bifidobacteria and butyrate-producing colon bacteria: Importance and strategies for their stimulation in the human gut. Front. Microbiol..

[18] Kassinen A., Krogius-Kurikka L., Mäkivuokko H., Rinttilä T., Paulin L., Corander J., Malinen E., Apajalahti J., Palva A. (2007). The fecal microbiota of irritable bowel syndrome patients differs significantly from that of healthy subjects. Gastroenterology.

[19] Rajilić–Stojanović M., Biagi E., Heilig H.G., Kajander K., Kekkonen R.A., Tims S., de Vos W.M. (2011). Global and deep molecular analysis of microbiota signatures in fecal samples from patients with irritable bowel syndrome. Gastroenterology.

[20] Pinto P.R., Mota I.F., Pereira C.M., Ribeiro A.M., Loureiro J.M., Rodrigues A.E. (2017). Separation and recovery of polyphenols and carbohydrates from Eucalyptus bark extract by ultrafiltration/diafiltration and adsorption processes. Sep. Purif. Technol..

[21] McDougall G.J., Shpiro F., Dobson P., Smith P., Blake A., Stewart D. (2005). Different polyphenolic components of soft fruits inhibit α-amylase and α-glucosidase. J. Agric. Food Chem..

[22] Kammerer D., Claus A., Carle R., Schieber A. (2004). Polyphenol screening of pomace from red and white grape varieties (*Vitis vinifera* L.) by HPLC-DAD-MS/MS. J. Agric. Food Chem..

[23] Liu Y., Wang S., Jin G., Gao K., Wang S., Zhang X., Zhou K., Cai Y., Zhou X., Zhao Z. (2023). Network pharmacology-based study on the mechanism of ShenKang injection in diabetic kidney disease through Keap1/Nrf2/Ho-1 signaling pathway. Phytomedicine.

[24] Sun Y., Zhong S., Yu J., Zhu J., Ji D., Hu G., Wu C., Li Y. (2018). The aqueous extract of Phellinus igniarius (SH) ameliorates dextran sodium sulfate-induced colitis in C57BL/6 mice. PLoS ONE.

[25] Chen Y., Xiao S., Gong Z., Zhu X., Yang Q., Li Y., Gao S., Dong Y., Shi Z., Wang Y. (2017). Wuji Wan formula ameliorates diarrhea and disordered colonic motility in post-inflammation irritable bowel syndrome rats by modulating the gut microbiota. Front. Microbiol..

[26] La J.-H., Kim T.-W., Sung T.-S., Kang J.-W., Kim H.-J., Yang I.-S. (2003). Visceral hypersensitivity and altered colonic motility after subsidence of inflammation in a rat model of colitis. World J. Gastroenterol. WJG.

[27] Williams C.L., Villar R.G., Peterson J.M., Burks T.F. (1988). Stress-induced changes in intestinal transit in the rat: A model for irritable bowel syndrome. Gastroenterology.

[28] Yu L., Huang C., Yang W., Ren Z., Li L., Cheng H., Lin C., Zhai L., Ning Z., Wong H.X. (2023). Aqueous cinnamon extract ameliorates bowel dysfunction and enteric 5-HT synthesis in IBS rats. Front. Pharmacol..

[29] Al–Chaer E.D., Kawasaki M., Pasricha P.J. (2000). A new model of chronic visceral hypersensitivity in adult rats induced by colon irritation during postnatal development. Gastroenterology.

[30] Yu Y., Wu S., Li J., Wang R., Xie X., Yu X., Pan J., Xu Y., Zheng L. (2015). The effect of curcumin on the brain-gut axis in rat model of irritable bowel syndrome: Involvement of 5-HT-dependent signaling. Metab. Brain Dis..

[31] Gao Z., He X., Zhao B., Zhou C., Liang Y., Ge R., Shen Y., Huang Z. (2010). Overexpressing a putative aquaporin gene from wheat, TaNIP, enhances salt tolerance in transgenic Arabidopsis. Plant Cell Physiol..

[32] Lippai D., Bala S., Csak T., Kurt-Jones E.A., Szabo G. (2013). Chronic alcohol-induced microRNA-155 contributes to neuroinflammation in a TLR4-dependent manner in mice. PLoS ONE.

[33] Nossa C.W., Oberdorf W.E., Yang L., Aas J.A., Paster B.J., DeSantis T.Z., Brodie E.L., Malamud D., Poles M.A., Pei Z. (2010). Design of 16S rRNA gene primers for 454 pyrosequencing of the human foregut microbiome. World J. Gastroenterol. WJG.

[34] Tzivion G., Dobson M., Ramakrishnan G. (2011). FoxO transcription factors; Regulation by AKT and 14-3-3 proteins. Biochim. Et Biophys. Acta (BBA) Mol. Cell Res..

[35] Zhang X., Tang N., Hadden T.J., Rishi A.K. (2011). Akt, FoxO and regulation of apoptosis. Biochim. Biophys. Acta (BBA) Mol. Cell Res..

[36] Cunningham D., Humblet Y., Siena S., Khayat D., Bleiberg H., Santoro A., Bets D., Mueser M., Harstrick A., Verslype C. (2004). Cetuximab monotherapy and cetuximab plus irinotecan in irinotecan-refractory metastatic colorectal cancer. N. Engl. J. Med..

[37] Ganesan S., Unger B.L., Comstock A.T., Angel K.A., Mancuso P., Martinez F.J., Sajjan U.S. (2013). Aberrantly activated EGFR contributes to enhanced IL-8 expression in COPD airways epithelial cells via regulation of nuclear FoxO3A. Thorax.

[38] Cory S., Adams J.M. (2002). The Bcl2 family: Regulators of the cellular life-or-death switch. Nat. Rev. Cancer.

[39] Rangel I., Sundin J., Fuentes S., Repsilber D., De Vos W., Brummer R.J. (2015). The relationship between faecal-associated and mucosal-associated microbiota in irritable bowel syndrome patients and healthy subjects. Aliment. Pharmacol. Ther..

[40] Bennet S.M., Öhman L., Simrén M. (2015). Gut microbiota as potential orchestrators of irritable bowel syndrome. Gut Liver.

[41] Lin X., Hu T., Wu Z., Li L., Wang Y., Wen D., Liu X., Li W., Liang H., Jin X. (2024). Isolation of potentially novel species expands the genomic and functional diversity of Lachnospiraceae. iMeta.

[42] Xiong L., Chen M., Chen H., Xu A., Wang W., Hu P. (2004). A population-based epidemiologic study of irritable bowel syndrome in South China: Stratified randomized study by cluster sampling. Aliment. Pharmacol. Ther..

[43] Lovell R.M., Ford A.C. (2012). Global prevalence of and risk factors for irritable bowel syndrome: A meta-analysis. Clin. Gastroenterol. Hepatol..

[44] Gao J., Xiong T., Grabauskas G., Owyang C. (2022). Mucosal serotonin reuptake transporter expression in irritable bowel syndrome is modulated by gut microbiota via mast cell–prostaglandin E2. Gastroenterology.

[45] Goodoory V.C., Tuteja A.K., Black C.J., Ford A.C. (2024). Systematic review and meta-analysis: Efficacy of mesalamine in irritable bowel syndrome. Clin. Gastroenterol. Hepatol..

[46] Chey W.D., Hashash J.G., Manning L., Chang L. (2022). AGA clinical practice update on the role of diet in irritable bowel syndrome: Expert review. Gastroenterology.

[47] Rej A., Sanders D.S., Shaw C.C., Buckle R., Trott N., Agrawal A., Aziz I. (2022). Efficacy and acceptability of dietary therapies in non-constipated irritable bowel syndrome: A randomized trial of traditional dietary advice, the low FODMAP diet, and the gluten-free diet. Clin. Gastroenterol. Hepatol..

[48] Wouters M.M., Van Wanrooy S., Nguyen A., Dooley J., Aguilera-Lizarraga J., Van Brabant W., Garcia-Perez J.E., Van Oudenhove L., Van Ranst M., Verhaegen J. (2016). Psychological comorbidity increases the risk for postinfectious IBS partly by enhanced susceptibility to develop infectious gastroenteritis. Gut.

[49] Lin S., Zhu Q., Wen L., Yang B., Jiang G., Gao H., Chen F., Jiang Y. (2014). Production of quercetin, kaempferol and their glycosidic derivatives from the aqueous-organic extracted residue of litchi pericarp with Aspergillus awamori. Food Chem..

[50] Lukšič L., Bonafaccia G., Timoracka M., Vollmannova A., Trček J., Nyambe T.K., Melini V., Acquistucci R., Germ M., Kreft I. (2016). Rutin and quercetin transformation during preparation of buckwheat sourdough bread. J. Cereal Sci..

[51] Wang Z., Ma J., Li X., Wu Y., Shi H., Chen Y., Lu G., Shen H., Lu G., Zhou J. (2020). Quercetin induces p53-independent cancer cell death via Tfeb-mediated lysosome activation and ros-dependent ferroptosis. Br. J. Pharmacol.

[52] Zheng X., Wu X., Wen Q., Tang H., Zhao L., Shi F., Li Y., Yin Z., Zou Y., Song X. (2023). Eriodictyol alleviated lps/D-galn-induced acute liver injury by inhibiting oxidative stress and cell apoptosis via pi3k/akt signaling pathway. Nutrients.

[53] Zumerle S., Sarill M., Saponaro M., Colucci M., Contu L., Lazzarini E., Sartori R., Pezzini C., Rinaldi A., Scanu A. (2024). Targeting senescence induced by age or chemotherapy with a polyphenol-rich natural extract improves longevity and healthspan in mice. Nat. Aging.

[54] Wei Y., Fan Y., Huang S., Lv J., Zhang Y., Hao Z. (2024). Baizhu shaoyao decoction restores the intestinal barrier and brain–gut axis balance to alleviate diarrhea-predominant irritable bowel syndrome via FoxO1/FoxO3a. Phytomedicine.

[55] Chang X., Zheng B., Guo Y., Chen Y., Xie J., Shan J., Wang Y., Xue P., Hu X., Hu X. (2024). Bound polyphenols in insoluble dietary fiber of navel orange peel modulate LPS-induced intestinal-like co-culture inflammation through CSF2-mediated NF-κB/JAK-STAT pathway. Food Funct..

[56] Kashyap P., Riar C.S., Jindal N. (2022). Effect of extraction methods and simulated in vitro gastrointestinal digestion on phenolic compound profile, bio-accessibility, and antioxidant activity of Meghalayan cherry (*Prunus nepalensis*) pomace extracts. Lwt.

[57] Reiter C.E., Kim J.-a., Quon M.J. (2010). Green tea polyphenol epigallocatechin gallate reduces endothelin-1 expression and secretion in vascular endothelial cells: Roles for AMP-activated protein kinase, Akt, and FOXO1. Endocrinology.

[58] Chen X., Peng B., Jiang H., Zhang C., Li H., Li Z. (2022). Salvianolic acid B alleviates oxidative stress in non-alcoholic fatty liver disease by mediating the SIRT3/FOXO1 signaling pathway. J. Chin. Pharm. Sci..

[59] Cardona F., Andrés-Lacueva C., Tulipani S., Tinahones F.J., Queipo-Ortuño M.I. (2013). Benefits of polyphenols on gut microbiota and implications in human health. J. Nutr. Biochem..

[60] Li S., Chen Y., Zhang Y., Lv H., Luo L., Wang S., Guan X. (2022). Polyphenolic extracts of coffee cherry husks alleviated colitis-induced neural inflammation via NF-κB signaling regulation and gut microbiota modification. J. Agric. Food Chem..

[61] Carasso S., Fishman B., Lask L.S., Shochat T., Geva-Zatorsky N., Tauber E. (2021). Metagenomic analysis reveals the signature of gut microbiota associated with human chronotypes. FASEB J..

